# Various steaming durations alter digestion, absorption, and fermentation by human gut microbiota outcomes of *Polygonatum cyrtonema* Hua polysaccharides

**DOI:** 10.3389/fnut.2024.1466781

**Published:** 2024-09-19

**Authors:** Weijing Wu, Yanling Wang, Ping Yi, Xufeng Su, Yan Mi, Lanlan Wu, Qianglai Tan

**Affiliations:** ^1^Xiamen Medical College, Xiamen, China; ^2^Engineering Research Center of Natural Cosmeceuticals College of Fujian Province, Xiamen Medical College, Xiamen, China; ^3^Fujian Provincial Key Laboratory of Food Microbiology and Enzyme Engineering, Xiamen, China

**Keywords:** *Polygonatum cyrtonema* Hua polysaccharides, steaming, saliva-gastrointestinal digestion, absorption, *in vitro* fermentation, gut microbiota

## Abstract

**Introduction:**

Different steaming durations dramatically alter the structure of *Polygonatum cyrtonema* polysaccharides (PCPs). This study aimed to compare characteristics of digestion, absorption, and fermentation by gut microbiota across four representative PCPs from different steaming durations (0, 4, 8, and 12 h), each with unique molecular weights and monosaccharide profiles.

**Methods:**

Chemical composition of the four PCPs was analyzed. Digestibility was evaluated using an *in vitro* saliva-gastrointestinal digestion model. Absorption characteristics were assessed with a Caco-2 monolayer model, and impacts on gut microbiota composition and short chain fatty acid (SCFA) levels were analyzed using *in vitro* fermentation with human gut microbiota.

**Results:**

Longer steaming durations altered the chemical profiles of PCPs, reducing carbohydrate content (84.87–49.58%) and increasing levels of uronic acid (13.99–19.61%), protein (1.07–5.43%), and polyphenols (0.05–2.75%). Four PCPs were unaffected by saliva digestion but showed enhanced gastrointestinal digestibility, with reducing sugar content rising from 4.06% (P0) to 38.5% (P12). The four PCPs showed varying absorption characteristics, with P0 having the highest permeability coefficient value of 9.59 × 10^−8^ cm/s. However, all PCPs exhibited poor permeability, favoring gut microbiota fermentation. The four PCPs altered gut microbiota composition and elevated SCFA production, but levels declined progressively with longer steaming durations. All PCPs significantly increased the abundance of Bacteroidota, Firmicutes, and Actinobacteriota, making them the dominant bacterial phyla. Additionally, all PCPs significantly increased the abundance of *Bifidobacterium, Prevotella*, and *Faecalibacterium* compared to the control group, which, along with *Bacteroides*, became the dominant microbiota. Increasing the steaming duration led to a reduction in *Prevotella* levels, with PCPs from raw rhizomes showing the highest relative abundance at 24.90%. PCPs from moderately steamed rhizomes (4 h) led to a significant rise in *Faecalibacterium* (7.73%) among four PCPs. P8 and P12, derived from extensively steamed rhizomes (≥8 h), exhibited similar gut microbiota compositions, with significantly higher relative abundances of *Bacteroides* (20.23–20.30%) and *Bifidobacterium* (21.05–21.51%) compared to P0 and P4.

**Discussion:**

This research highlights the importance of adjusting steaming durations to maximize the probiotic potential of *P. cyrtonema* polysaccharides, enhancing their effectiveness in modulating gut microbiota and SCFA levels.

## 1 Introduction

The rhizomes of *Polygonatum cyrtonema* Hua have been utilized in Chinese functional foods for centuries, known for their broad biological effects (1–5). *P. cyrtonema* is effective for treating conditions such as fatigue, diabetes, colitis and enhancing immune activity, as well as possessing hypolipidemic properties (4, 6–14). According to traditional Chinese medicine principles, *P. cyrtonema*’s nourishing qualities are thought to bolster and fortify the gastrointestinal tract. Modern research supports these traditional uses by linking the effects to *P. cyrtonema* polysaccharides (PCPs). As macromolecules, these polysaccharides typically aren’t fully digested or absorbed; instead, they serve as nourishment for the gut microbiota. This interaction between the polysaccharides and gut microbiota is critical to their physiological effectiveness, highlighting the integral role of microbial health in leveraging the potential of PCPs.

However, the raw rhizomes often cause throat and tongue irritation when consumed directly, so steaming has become an essential processing step prior to use (2, 8, 15–19). This process dramatically alters the structural characteristics, affecting their molecular weight, particle size, and monosaccharide composition. Our prior studies have shown that moderate steaming lengths (2–4 h) promote PCPs aggregation, resulting in higher molecular weights and particle sizes compared to those from raw rhizomes. Steaming also altered the monosaccharide composition of PCPs (6, 12, 20, 21). The major monosaccharide in PCPs that from rhizomes steamed for 2 and 4 h was xylose, while the PCPs from raw rhizomes were mainly composed of fructose (20). However, extensive steaming durations of 6–12 h degrade these polysaccharides which are mainly compose of galactose, adversely affecting their immune-modulating properties (21). The *in vivo* immune activity of PCPs varied significantly with steaming frequency of the steaming process (8). Since structural characteristics support the function and bioactivity of polysaccharides (22), the duration of steaming, which alters the structural characteristics of PCPs, is a critical factor influencing their activities.

Furthermore, salivary amylase, the severe pH in the gastric fluid, pancreatin and bile acids in the gastrointestinal tract may break down the glycosidic bonds of polysaccharides (23), influencing their the fermentation properties by gut microbiota (24). It was shown that polysaccharides with different molecular weights have varying effects on gut microbiota fermentation (24). The PCPs from different steaming durations had varying molecular weight. Although the neutral PCPs from raw rhizomes have shown the various effects (2), the structural changes of PCPs from steamed rhizomes and their interactions with the gut microbiota are still not fully understood, particularly how different steaming durations affect these interactions.

Thus, building on previous findings, this study focuses on four representative steaming durations (0, 4, 8, and 12 h), representing the raw rhizomes, aggregation of PCPs, destruction of newly formed PCPs aggregates, and excessive destruction of PCPs aggregates. These four PCPs display significant variations in molecular weight and monosaccharide composition. This research aims to elucidate the chemical composition changes of PCPs induced by varying steaming durations, particularly in relation to their *in vitro* gastrointestinal digestion, absorption, and fermentation characteristics, especially their interaction with gut microbiota.

## 2 Materials and methods

### 2.1 Material and reagents

*P. cyrtonema* rhizomes (wild-type) were sourced from Shaowu, Fujian, China. High-purity short-chain fatty acids (SCFAs) (≥99.5%) were acquired from Aladdin Company, Shanghai, China. Enzymes such as gastric lipase, pepsin (≥250 U/mg), pancreatin, and trypsin (≥250 U/mg) were obtained from Sigma, China. All additional chemicals were of analytical grade.

### 2.2 Processing of *P. cyrtonema*, extraction, and chemical analysis of PCPs

The method for processing and extracting PCPs followed protocols established in our previous research (21). Initially, the dried rhizome slices were steamed for 4, 8, and 12 h and subsequently dried at 55°C overnight. The samples were ground and subjected to defatting using 95% ethanol (v/v) at 80°C for 1 h. The defatted materials were then extracted with distilled water (1:10, w/v) with stirring at 80°C for 1 h. The resulting crude polysaccharides were isolated via filtration and subsequent precipitation with four volumes of anhydrous ethanol. The precipitated polysaccharides were redissolved in water, stirred for 2 h, and the insoluble particles were eliminated through centrifugation at 10,000 r/min for 15 min. The supernatant was freeze-dried to obtain the final polysaccharide products, designated as P4, P8, and P12 for the steamed variants, and P0 for the raw rhizomes. Following the previous study, the chemical composition of PCPs was analyzed with some modifications (25).

#### 2.2.1 Total carbohydrate content

This was quantified using the phenol/sulfuric acid colorimetric method (26). Briefly, 0.4 mL of the sample was mixed with 6% phenol solution and 1 mL sulfuric acid. The absorbance was measured at 490 nm, with glucose used for the calibration curve, ranging from 0 to 100 μg/mL.

#### 2.2.2 Uronic acid content

The concentration of uronic acids in the samples was determined using the m-hydroxybiphenyl method, with galacturonic acid as the standard, ranging from 0 to 200 μg/mL.

#### 2.2.3 Protein content

Protein content was measured using the Coomassie Brilliant Blue method with bovine serum albumin (BSA) as the standard, within a range of 0 to 0.5 mg/mL. Additionally, ultraviolet–visible (UV) spectrophotometry was employed to detect nucleic acids (absorbance peak at 260 nm) and proteins (absorbance peak at 280 nm) in the PCPs. The polysaccharide and BSA solutions, each at a concentration of 0.5 mg/mL, scanned over a wavelength range of 200–400 nm.

#### 2.2.4 Polyphenol content

Polyphenol content in the samples was analyzed by the Folin–Ciocalteu assay using gallic acid as the standard, with a range from 0 to 0.07 mg/mL. Briefly, 0.5 mL of the sample was mixed with an equal amount of 0.25 mol/L Folin phenol reagent, followed by the addition of 1 mL of 15% Na₂CO_3_. After 30 min, the mixture centrifuged at 3500 r/min, and the supernatant was measured at 760 nm.

### 2.3 *In vitro* saliva-gastrointestinal digestion of PCPs

#### 2.3.1 Simulation of *in vitro* saliva-gastrointestinal digestion process for PCPs

The *in vitro* digestion of PCPs was adapted from established protocols with minor modifications (27–29). Salivary juice was collected from four healthy volunteers, who had not received antibiotics or experienced any infectious diseases within the past 3 months. Volunteers fasted for 2 h prior to collection, and their mouths were rinsed with water. Saliva was collected every 2 min, combined from all volunteers, and centrifuged at 5,000 r/min at 4°C for 10 min to obtain the supernatant for use in experiments. The PCPs solution (2 mg/mL) was mixed with an equal volume of this saliva and incubated at 37°C with stirring. Samples were taken at intervals of 0, 2, and 4 h, immediately boiled for 5 min to stop enzymatic activity, and stored at −20°C for subsequent analysis. This process was replicated three times for consistency.

Simulated gastric juice was created by combining 150 mg of gastric lipase, 141.6 mg of pepsin, and 2 mL of CH_3_COONa solution (1 mol/L, pH 5.0) with 1 L of gastric electrolyte solution (comprising 3.1 g/L NaCl, 1.1 g/L KCl, 0.6 g/L NaHCO_3_, and 0.15 g/L CaCl_2_, pH 3.0), and then adjusting the mixture to pH 3.0. The PCPs solution (10 mg/mL) was mixed with an equal volume of this gastric juice and digested at 37°C with stirring at 150 r/m, maintaining a pH of 3.0. Samples were collected at 0, 2, and 4 h, immediately boiled for 5 min to inactivate enzymes for analysis.

Artificial small intestinal juice was prepared by mixing 100 mL artificial intestinal electrolyte solution (5.4 g/L NaCl, 0.65 g/L KCl, 0.3 g/L NaHCO_3_, and 0.25 g/L CaCl_2_, pH 7.0), 200 mL bile salt solution (4% w/w), 100 mL pancreatin solution (7% w/w), and 13.0 mg trypsin to reach a final pH of 7.5. The gastric-digested sample was then mixed with this intestinal juice in a 10:3 volume ratio and incubated at 37°C with stirring at 150 r/min. The pH was maintained at 7.5, and samples were collected at designated times, boiled for enzyme inactivation, and prepared for further testing.

Samples were centrifuged, and the supernatants were split into two portions for different analyses. One part was used for reducing sugar measurement and molecular weight determination. The other part was precipitated with four volumes of anhydrous ethanol and lyophilized for scanning electron microscopy.

#### 2.3.2 Reducing sugar content analysis

Reducing sugar levels were quantified using the 3,5-dinitrosalicylic acid (DNS) method, with glucose serving as the standard (30).

#### 2.3.3 Molecular weight of PCPs

According to a previous study (31), the molecular weight distribution of the PCPs was assessed using high-performance gel permeation chromatography (HPGPC) coupled with multi-angle laser light scattering and a differential refractive index detector (OPTILAB DSP, Wyatt Technology Inc., United States).

The samples were eluted with 0.1 M sodium nitrate solution at a flow rate of 0.5 mL/min. The concentration of each eluted fraction was determined by differential refractive index according to the known value of dN/dC = 0.15. The data were analyzed by using Astra software (version 5.3.4, Wyatt).

#### 2.3.4 Scanning electron microscopy analysis

For SEM analysis, 0.2 mg of dried PCPs was mounted on a sample stage and coated with a thin layer of gold using Magnetron sputter. The morphological characteristics of the PCPs were observed under a scanning electron microscope (FlexSEM 1000, Hitachi Ltd., Japan) at 7 kV and a magnification of 500×.

### 2.4 Absorption of FITC (fluorescein isothiocyanate)-labeled PCPs by Caco-2 monolayer model

#### 2.4.1 Preparation of PCP-TYR (tyramine)-FITC

The conjugation of PCPs with TYR and further labeling with FITC was conducted by adapting methodologies from prior study (32). Initially, 400 mg of various PCPs were dissolved in 15 mL of phosphate buffer (0.2 mol/L, pH 8.0). Then, 400 mg of tyramine and 150 mg of sodium cyanoborohydride were added to this solution. The mixture was incubated at 37°C for 96 h with shaking. It was centrifuged at 10,000 r/min for 10 min, the supernatant was collected, freeze-dried, and its UV–VIS spectrum was scanned from 200 to 350 nm.

Subsequently, the PCP-TYR was labeled with FITC. PCP-TYR was dissolved in 10 mL Na_2_CO_3_ solution (0.2 mol/L, pH 8.5), to which 25 mg of FITC dissolved in 50 μL DMSO was added. The mixture was stirred at room temperature for 24 h shielded from light. Following this, the reaction mixture was precipitated with four volumes of anhydrous ethanol, centrifuged, and the precipitate was washed until the supernatant showed no absorbance at 490 nm. The PCP-TYR-FITC was then re-dissolved in water and freeze-dried. The degree of FITC substitution was calculated based on fluorescence intensities at an excitation wavelength of 490 nm and an emission wavelength of 515 nm, compared against a standard FITC curve.

#### 2.4.2 Cell culture

Caco-2 cells (SCSP-5027) were purchased from the national collection of authenticated cell cultures and cultured in Dulbecco’s modified Eagle’s medium (DMEM) supplemented with 10% fetal bovine serum, 100 IU/mL Penicillin, and 100 μg/mL streptomycin. The cells were maintained at 37°C in a humidified 5% CO_2_ atmosphere.

#### 2.4.3 Cytotoxicity assays of FITC-labeled PCPs

According to previous studies (33, 34), Caco-2 cell viability was assessed using the colorimetric CCK-8 assay. Cells were seeded in 96-well plates at a density of 2 × 10^4^ cells/well and incubated overnight for attachment to the bottom. Samples were prepared with medium and added to the wells. Cells were exposed to final concentrations of 0, 0.2 mg/mL and 1 mg/mL for 4 h and 24 h. Subsequently, the medium was discarded and replaced with 100 μL fresh medium containing CCK-8, and the cells were incubated for another 2 h. Absorbance was measured at 450 nm using a microplate reader (Infinite M200, Tecan, Austria), with each concentration tested in quadruplicate.

#### 2.4.4 Construction and evaluation of Caco-2 cells monolayer model

The construction of the Caco-2 monolayer was conducted according to a previous studies (34, 35). Caco-2 cells were seeded into 12-well polyester Transwell plates at a density of 2 × 10^5^ cells/well. The medium in the apical (AP) side was changed every 2 days, and in the basolateral (BL) side every 4 days. Monolayers with transepithelial electrical resistance (TEER) values above 600 *Ω*/cm^2^ (Millicell-ERS, Millipore, Bedford, United States) were used for transport studies. Alkaline phosphatase (AKP) activity in AP and BL compartments and the transport of sodium fluorescein (10 μg/mL) was assessed to further confirm cell monolayer integrity and membrane functionality.

#### 2.4.5 Absorption of FITC-labeled PCPs

Absorption of FITC-labeled PCPs across Caco-2 monolayers was evaluated following established protocols (34, 35). After 21 days of monolayer maturation, 500 μL of a 1,000 μg/mL solution of FITC-labeled PCPs was added to the AP side. The BL side was refreshed with 1.5 mL of new medium. A 200 μL sample was taken from the BL side to 96-well black plate at designated time intervals. The same amount of fresh medium was supplemented in the BL side. The fluorescence intensity in the BL side was measured at excitation and emission wavelengths of 490 nm and 515 nm, respectively. The reference standard of different FITC-labeled PCPs was prepared at range of 1.25–50 μg/mL. The apparent permeability coefficient (P*app*) value was calculated using the following equation: P*app* = (dQ/dt) / (A × C_0_), where dQ/dt represents the transport rate, A is the area of the membrane (1.12 cm^2^), and C_0_ is the initial FITC-labeled PCPs (1,000 μg/mL).

### 2.5 *In vitro* fecal fermentation by human gut microbiota

#### 2.5.1 Preparation of fecal inoculum and fermentation setup

The *in vitro* fecal fermentation process was adapted from established protocols with minor modifications (36). The fermentation medium, prepared in 1.0 L, consisted of 7.2 g of ET media, 2.0 mL of Tween 80, 0.025 g of resazurin, 10 μL of vitamin K, and 0.02 g of hemin, and was sterilized at 121°C for 30 min. Fresh fecal samples were collected from seven healthy volunteers (three males and four females, aged 20–35 years), who had no digestive diseases or antibiotic treatments within the last 3 months. The collected fecal matter was homogenized with cold phosphate-buffered saline (PBS) in a 1:4 (w/v) ratio and then filtered through a 250 μm mesh using a homogenization bag (Bagfilter P400, Interscience) to create a fecal slurry. Eight milliliters of the fecal slurry were mixed with 32 mL of basal nutrient growth medium enriched with 400 mg of different PCPs or fructo-oligosaccharides (FOS). FOS served as a positive control. This mixture was transferred to an anaerobic chamber. The fermentation process was monitored over a 24-h period, with samples collected at 0, 6, 12, and 24 h. The fermentation samples designated for microbiota analysis were immediately frozen and stored at −80°C. The remaining portions of the samples were boiled for 5 min to halt the fermentation process. Three replicates were conducted for each group.

#### 2.5.2 Analysis of fermentation products

The supernatant from fermentation product that centrifugated at 8,000 r/min for 15 min, was used for further measurement to assess the metabolic activity of the microbiota:

##### 2.5.2.1 pH measurement

The pH of each fermentation sample was recorded.

##### 2.5.2.2 SCFAs quantification

Based on our previous research (37), SCFAs in the fermentation supernatants were analyzed via gas chromatography with a flame ionization detector (7890B GC system, Agilent, United States). The injection port was operated at 250°C, with an injection volume of 1 μL. Following filtration of the supernatant through a 0.22 μm filter, SCFAs were separated on an HP INNO-WAX column (ID 25 mm, length 30 m, 0.25 μm film thickness, JW scientific, United States). The temperature program started at 100°C, ramped up to 125°C at 3°C/min, increased to 200°C at 20°C/min, and was held at 230°C for 2 min. The detector’s temperature was maintained at 280°C, with nitrogen as the carrier gas at a flow rate of 25 mL/min. Quantification was based on standards detailed in the Supplementary Table S1.

In addition, total carbohydrate, uronic acid, and reducing sugar contents were measured as previously described in Section 2.2.

#### 2.5.3 Microbiota composition analysis by 16 s rRNA sequencing

Microbial community composition was assessed through 16S rRNA Sequencing. Bacterial DNA was extracted using a MagPure Soil DNA LQ Kit and assessed for concentration and integrity via using a NanoDrop 2000 spectrophotometer and agarose gel electrophoresis. PCR amplification targeted the V3-V4 hypervariable regions of the 16S rRNA gene using specific primers, with sequencing adapters and sample barcodes included. PCR products were purified, quantified, and prepared for sequencing on an Illumina NovaSeq 6000 platform. Sequencing and data analysis were performed by OE Biotech Co., Ltd. (Shanghai, China).

### 2.6 Statistical analysis

The data are expressed as means with standard deviations (SD). Statistical differences among groups were evaluated by one-way analysis of variance (ANOVA) and Duncan’s test for *post hoc* analysis. Data were considered statistically significant at *p* < 0.05.

## 3 Results and discussion

### 3.1 Chemical composition analysis of PCPs

Analysis of P0, P4, P8, and P12 revealed significant variations in their chemical compositions. The total carbohydrate content decreased progressively from P0 to P12, with values of 84.87 ± 4.58% for P0, 59.82 ± 1.00% for P4, 48.25 ± 1.82% for P8, and 49.58 ± 1.18% for P12. In contrast, the uronic acid content increased across these samples, with P0 at 13.99 ± 2.74%, P4 at 19.36 ± 2.64%, P8 at 19.57 ± 1.55%, and P12 at 19.61 ± 1.58%, corresponding to pH values of the polysaccharide solutions of 5.53, 4.54, 4.23, and 3.93, respectively. Additionally, protein content showed a marked increase, with P0 at 1.07 ± 0.32%, P4 at 1.12 ± 0.31%, P8 at 2.64 ± 0.11%, and P12 at 5.43 ± 0.12%. The absence of prominent absorption peaks at 260 nm and 280 nm in the UV spectrogram (Supplementary Figure S1) indicates the low content of protein and nucleic acid in PCPs. Finally, polyphenol content also varied, starting from 0.05 ± 0.01% in P0, rising to 0.47 ± 0.02% in P4, peaking at 2.75 ± 0.29% in P8, and slightly decreasing to 2.23 ± 0.08% in P12. These changes in chemical composition are consistent with trends observed in PCPs processed by the nine-steam-nine-bask method, where total carbohydrate content decreased, protein content increased, and uronic acid content significantly rose (38). In previous studies, the total carbohydrate content in polysaccharides from *P. cyrtonema* was found to be 70.31% (39). Additionally, polyphenol content in *P. cyrtonema* rhizomes increased 5.57-fold during processing (40). Although primary free polyphenols were removed by ethanol before polysaccharide extraction, interactions between polysaccharides and other compounds, forming complexes under specific conditions, could be selectively triggered during processing, leading to the retention of polyphenols within the polysaccharide (25). This contributed to the increase in polyphenol content in PCPs. In summary, these chemical composition changes reflect the impact of steaming duration on the structural properties of PCPs, which could influence their biological activities.

### 3.2 Dynamic changes of structural characterizations of different PCPs during *in vitro* saliva-gastrointestinal digestion

#### 3.2.1 Digestibility of different PCPs

The digestibility of various PCPs was evaluated by *in vitro* saliva-gastrointestinal digestion, focusing on the influence of steaming duration on the breakdown of glycosidic linkages within these polysaccharides. Table 1 presents the changes in reducing sugar content, which serves as an indicator of glycosidic linkage breakdown. An increase in steaming duration correlated with higher initial reducing sugar content, rising from 0.0170 mg/mL (P0) to 0.1586 mg/mL (P12). This trend suggests enhanced breakdown of glycosidic linkages as a result of prolonged steaming, corroborating findings from our previous research (21). The salivary digestion phase showed no significant change in reducing sugar levels across all PCP types (Table 1), indicating the resistance of PCPs to breakdown by salivary amylase, which primarily hydrolyzes *α*-(1 → 4)-glycosidic bonds in starchy polysaccharides (41). Similar resistance to α-amylase has also been observed in polysaccharides from *P. cyrtonema*, *Ziziphus jujuba* cv. *Pozao*, rapeseed, and *Cissus quadrangularis* (39, 42–44).

**Table 1:** Changes of reducing sugar contents (mg/mL) during *in vitro* simulated saliva-gastrointestinal digestion of PCPs.

	Digestion time	P0	P4	P8	P12
Saliva digestion	0 h	0.0170 ± 0.0086^a^	0.0559 ± 0.0139^a^	0.1235 ± 0.0187^a^	0.1586 ± 0.0091^a^
	2 h	0.0191 ± 0.0002^a^	0.0621 ± 0.0051^a^	0.1306 ± 0.0060^a^	0.1608 ± 0.0137^a^
	4 h	0.0165 ± 0.0014^a^	0.0669 ± 0.0149^a^	0.1292 ± 0.0059^a^	0.1668 ± 0.0076^a^
Gastric digestion	0 h	0.0820 ± 0.0019^b^	0.2976 ± 0.0418^b^	0.3881 ± 0.0194^b^	0.5112 ± 0.0239^b^
	2 h	0.1374 ± 0.0067^c^	0.3374 ± 0.0151^b^	0.3844 ± 0.0119^b^	0.4927 ± 0.0326^b^
	4 h	0.1392 ± 0.0044^c^	0.3955 ± 0.0120^bc^	0.4561 ± 0.0032^c^	0.5829 ± 0.0186^b^
Intestinal digestion	0 h	0.1471 ± 0.0100^c^	0.3721 ± 0.0843^bc^	0.5071 ± 0.0409^c^	1.0850 ± 0.0653^c^
	2 h	0.1549 ± 0.0252^c^	0.5032 ± 0.0511^cd^	0.4753 ± 0.0146^c^	1.3554 ± 0.0682^d^
	4 h	0.1559 ± 0.0036^c^	0.5549 ± 0.0195^d^	0.6807 ± 0.0011^d^	1.4894 ± 0.0340^e^

^*^Different letter indicated significant difference within in the same PCP group (*p* < 0.05).

During the gastric digestion phase, a marked increase in reducing sugar content was observed for all PCP groups, likely due to the acidic conditions of gastric acid, which play a crucial role in the decomposition of glycosidic bonds and the formation of reducing ends in polysaccharides. Notably, the P0 group (unsteamed PCPs) exhibited significant changes during the second hour of gastric digestion but showed no change during the intestinal digestion phase, consistent with a previous study (39). In contrast, the steamed PCP groups (P4, P8, and P12) displayed significant changes primarily during intestinal digestion, with the P12 group showing the most substantial increase in digestibility, particularly at the 2-h and 4-h marks. A significant increase in reducing sugar content during the gastric phase was observed in various polysaccharides with different molecular weights, where the significant changes most likely occurred due to the addition of gastric acid. These polysaccharides include polysaccharides from *Ziziphus jujuba* cv. *Pozao* with a high molecular weight of 1298.85 kDa (42), a pectic polysaccharide from okra with a molecular weight of 224.2 kDa (45), polysaccharides from *Clitocybe squamulose* with a molecular weight of 19.48 kDa (46), polysaccharides from longan pulp with molecular weights of 221.63 kDa and 109.62 kDa (47), and polysaccharides from *P. cyrtonema* with a molecular weight of 5.73 kDa (39), although most polysaccharides are resistant to degradation in the stomach (41). In comparison, the reducing sugar content did not significantly change in intestinal phase, such as observed in the polysaccharides from *Ziziphus jujuba cv. Pozao* (42) and a pectic polysaccharide from okra (45). However, changes in reducing sugar especially for 2^nd^ hour were found in polysaccharides of oyster (48).

In conclusion, the final reducing sugar content in PCPs after complete saliva-gastrointestinal digestion were 4.06% for P0, 14.4% for P4, 17.7% for P8, and 38.5% for P12. These findings suggest that longer steaming durations significantly enhance the digestibility of PCPs, resulting in more digestible polysaccharides during gastrointestinal digestion.

#### 3.2.2 Molecular weight changes of PCPs during *in vitro* gastrointestinal digestion

**Figure 1:**
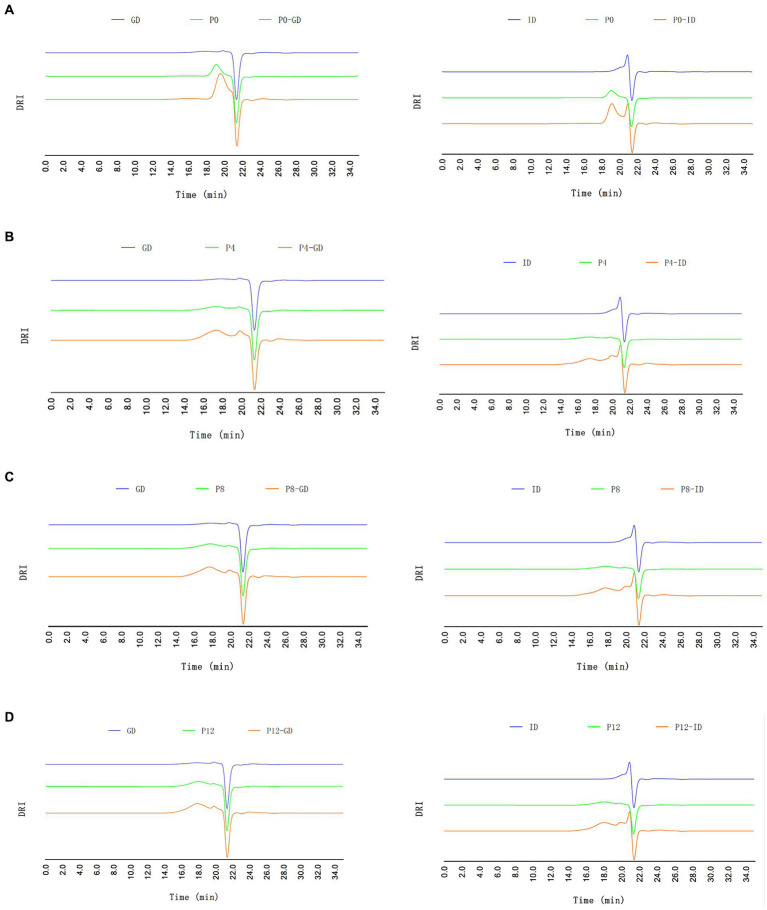
HPGPC-RID profiles of different PCPs during gastric digestion (GD) and intestinal digestion (ID) processes. **(A)** P0, **(B)** P4, **(C)** P8, **(D)** P12.

Given the limited changes of PCPs during the saliva digestion, the changes in molecular weight of PCPs during gastrointestinal digestion were further investigated, as shown in Figure 1. Before digestion, PCPs derived from steamed rhizomes exhibited a higher molecular weight compared to those from raw rhizomes, which was consistent with our previous study (21). In line with the results for reducing sugar content, PCPs from raw rhizomes (P0) showed a noticeable decrease in molecular weight occurred after 4 h of gastric digestion, but remained stable during the intestinal phase, indicating that the primary structural breakdown happened in the gastric phase (Figure 1A). Previous studies reported that polysaccharides with a higher molecular weight of 1298.85 kDa experienced a lower degree of molecular reduction (9.6%) compared to polysaccharides with a smaller molecular weight (19.48 kDa), which showed reductions of 47.68% after saliva-gastrointestinal digestion (42, 46). This is consistent with the significant reduction in molecular weight observed during the gastric digestion phase in P0, which had the lowest molecular weight fraction among the PCPs. In contrast, PCPs from steamed rhizomes (Figures 1B–D) underwent significant changes during intestinal digestion, forming lower molecular weight polysaccharides, aligning with the increase in reducing sugar content observed in section 3.2.1. This suggests a more extensive breakdown of polysaccharide chains during the intestinal phase, likely due to structural alterations caused by the steaming process. It confirms that the gastrointestinal digestion phase plays a crucial role in further decomposing the polysaccharides, particularly those pre-treated by steaming, as they are presumably more susceptible to enzymatic action due to their altered physical and chemical properties.

#### 3.2.3 SEM analysis of PCPs during *in vitro* gastrointestinal digestion

**Figure 2:**
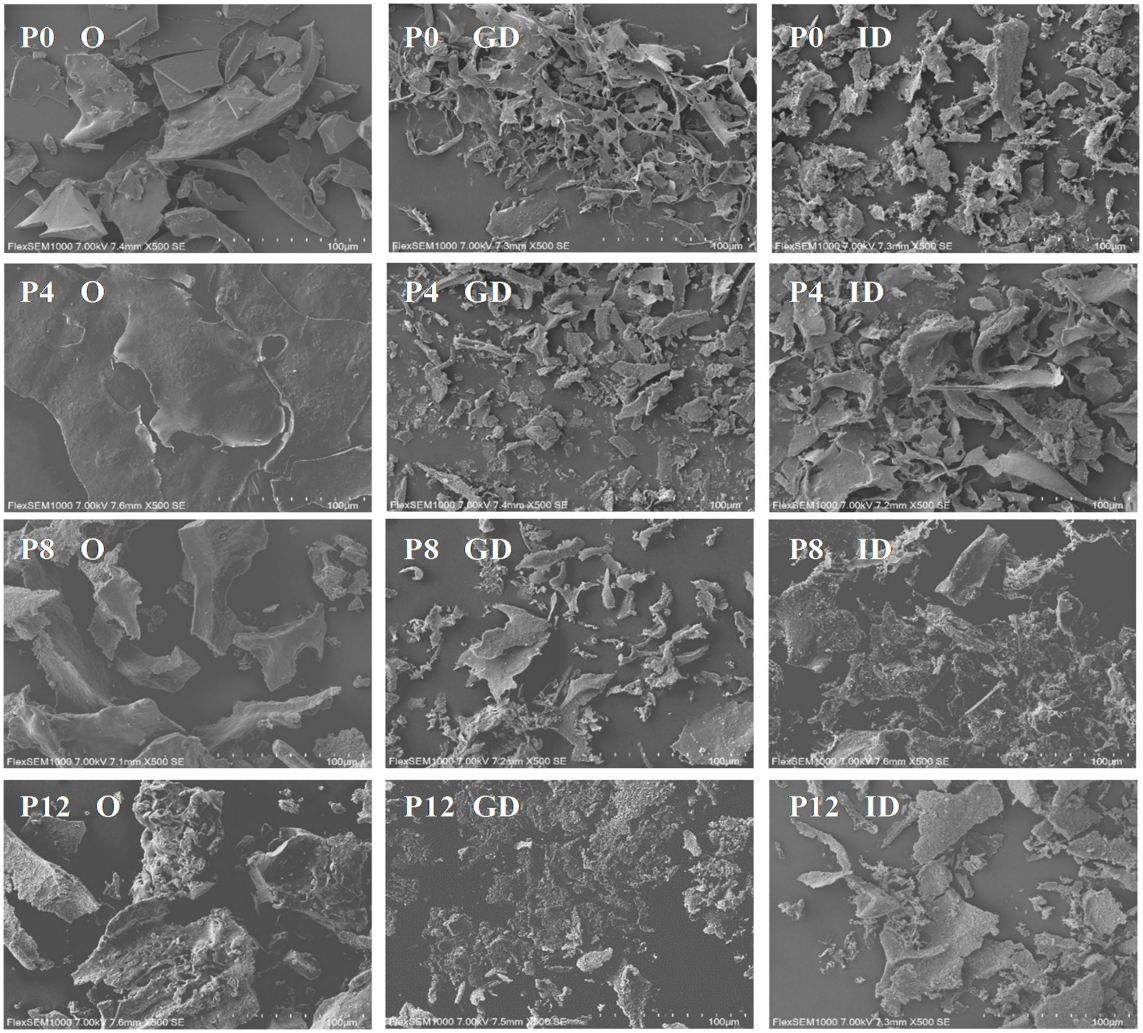
Scanning electron microscopy reveals structural changes in PCPs during gastric and intestinal digestion (O, original PCPs; GD, gastric digestion; ID, intestinal digestion).

SEM analysis provides insight into the surface morphology of PCPs during *in vitro* gastrointestinal digestion, as captured in Figure 2. The PCPs from raw rhizomes (P0) and those steamed for shorter durations (P4) initially displayed smooth flakes, with P4 exhibiting a smooth sheet-like structure due to its higher molecular weight (21). As the steaming duration increased, more significant surface erosion and distinct hole formation within a flaky structure were observed in P8 and P12, likely due to the degradation of PCPs caused by the steaming process. This suggests that the longer steaming durations contribute to a looser, more porous structure, likely enhancing the accessibility of digestive enzymes to the polysaccharide chains. After gastric digestion, all PCP samples displayed irregular fragments. This fragmentation suggests that gastric conditions, primarily the acidic environment, initiate the breakdown of glycosidic bonds within the PCPs. The intestinal phase of digestion further altered the PCPs’ morphology, with the formation of smaller pores across the samples. The observed porous structure in the PCPs correlates with reducing sugar content and molecular weight changes, resulting in a rough, cracked surface and irregular appearance, likely due to the breakdown of glycosidic bonds (49, 50). Similar results have also been observed with polysaccharides from *Clitocybe squamulose* (46) and *Ziziphus jujuba* cv. *Pozao* (42), where the shape and surface morphology changed significantly after gastrointestinal digestion, exhibiting irregular fragments.

In conclusion, PCPs was partially digested in gastric and gastrointestinal digestion. Different structure caused by steaming led to significant difference in digestive patterns, which could further affect their activities.

### 3.3 *In vitro* absorption of FITC labeled-PCPs by Caco-2 monolayer model

#### 3.3.1 FITC labeled PCPs

**Figure 3:**
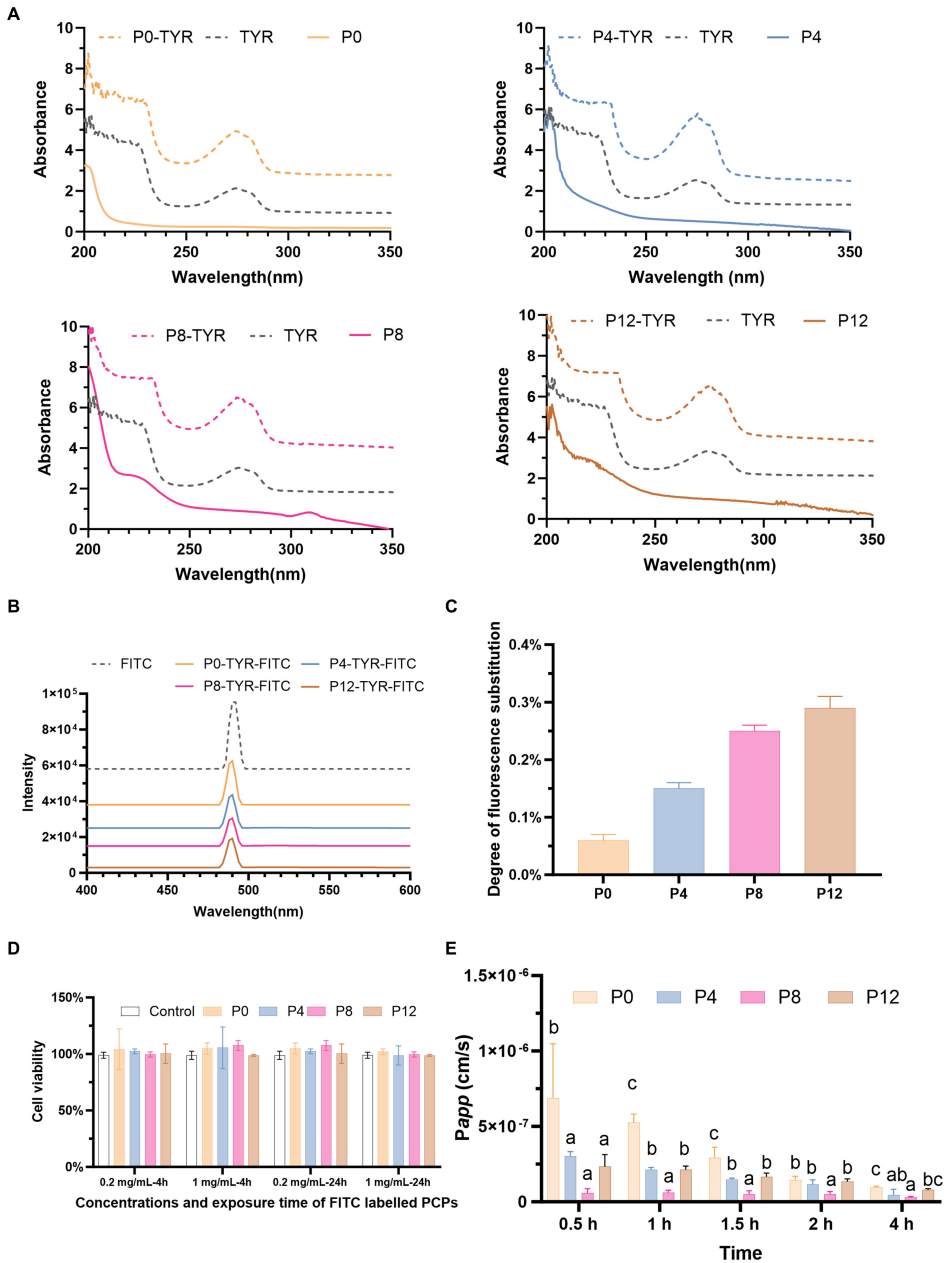
PCP-TYR-FITC labeling process and associated biological assays: **(A)** Comparative UV spectra of PCP, PCP-TYR, and TYR. **(B)** UV spectra of different PCPs-TYR-FITC. **(C)** Fluorescence substitution levels of different PCPs. **(D)** Cytotoxicity of FITC-labeled PCPs incubated for 4 h and 24 h on Caco-2 cells. **(E)** Permeability (P*app*) values of different FITC-labeled PCPs from AP to BL.

Due to the lack of direct polysaccharide detection methods in absorption studies using a Caco-2 monolayer model, the PCPs were first chemically modified with TYR and subsequently conjugated with FITC, enabling fluorescent labeling for detection. The labeling of TYR and FITC on PCPs was analyzed by fluorescence spectroscopy. The successful conjugation of PCPs with TYR was confirmed by the appearance of characteristic absorption peaks at 280 nm, corresponding to TYR, compared to the PCPs alone (Figure 3A). Figure 3B showed the successful conjugation of FITC to PCPs-TYR with a typical absorption peak at 490 nm for FITC. These indicate effective FITC linkage to the PCPs which can be used in subsequent experiments, with varying degrees of fluorescence substitution ranging from 0.06% for P0, 0.15% for P4, 0.25% for P8, and 0.29% for P12, as demonstrated in Figure 3C. A similar labeling process was applied to polysaccharides from *Cucurbita moschata* and *Codonopsis Radix* (33, 34).

#### 3.3.2 Toxicity of FITC-labeled PCPs in Caco-2

Figure 3D illustrates the cytotoxicity assessment of FITC-labeled PCPs at concentrations of 0.2 mg/mL and 1 mg/mL after incubation for both 4 h and 24 h. The results showed that no significant toxicity at these concentrations, demonstrating the low toxicity of FITC-PCPs. This finding is consistent with the long-standing recognition of *P. cyrtonema* as a food and medicinal material used to promote health for centuries. Based on this, 1 mg/mL FITC-PCPs was selected for subsequent transport studies which lasted for 4 h. Consistent with, the *Codonopsis Radix* polysaccharide at 1 mg/mL showed no toxicity, making it suitable transport studies (34).

#### 3.3.3 Establishment of Caco-2 monolayer model for cellular uptake of PCPs

The Caco-2 cell monolayer model is widely used to study nutrient transport across the intestinal barrier (33, 34). As shown in Supplementary Figure S2A, Caco-2 cells reached confluence and formed a monolayer by day 14. The TEER value, indicative of epithelial barrier integrity, increased over time and reached 618.7 *Ω* × cm^2^ by day 21, compared to 126.9 Ω × cm^2^ in blank control well without cells (Supplementary Figure S2B). This result is consistent with previous studies, which suggest that monolayers with TEER values exceeding 500 Ω·cm^2^ are suitable for transport experiments (33, 34). Furthermore, AKP activity was significantly higher on AP side than BL side (Supplementary Figure S2C), indicating proper cellular polarity and functionality. Additionally, the permeability of sodium fluorescein remained below 5.0 × 10^−7^ cm/s at all time points (Supplementary Figure S2D), confirming the monolayer’s integrity (33, 34). Thus, the successful formation of the monolayer was validated by TEER, AKP activity in the AP/BL compartments, and sodium fluorescein permeability for further transport studies.

#### 3.3.4 Absorption characteristics of FITC-labeled PCPs

Transport studies are typically conducted over a 4-h period (33, 35). As shown in Figure 3E, different PCPs exhibited varying uptake characteristics. The P*app* values for all PCPs remained below 1.0 × 10^−6^ at all time points, meeting the criteria for poor absorption (51). Our findings align with prior research, which also demonstrated that *Polygonatum sibiricum* polysaccharide has poor absorption and low bioavailability in rats (32). However, previous studies have demonstrated contrasting absorption characteristics of polysaccharides with different molecular weights. Polysaccharides with small molecular weights of 12 kDa and even as high as 108 kDa can be moderately well-absorbed (35, 52). In contrast, chitosan with higher molecular weights, especially between 100 kDa and 400 kDa, has shown no detectable permeation (53). Our previous study found that all PCPs had relatively high molecular weights, ranging from 334.8 kDa (P0), 1,163 kDa (P4), 112.4 kDa (P8), 67.1 kDa (P12) (21), which may contribute to their low absorption.

In addition, at the 4-h time point, the P*app* values were 9.59 × 10^−8^ cm/s for P0, 4.41 × 10^−8^ cm/s for P4, 2.89 × 10^−8^ cm/s for P8, and 7.82 × 10^−8^ cm/s for P12, respectively. Among the four PCPs, P0, which had fraction with the smallest molecular weight of 7.074 kDa (21), exhibited the highest absorption at all time points compared to the PCPs from steamed rhizomes (P4, P8, and P12). Consistent with our finding, polysaccharides from *Codonopsis Radix* with molecular weight as low as 3 kDa have shown absorption difficulties, with P*app* values below 1.0 × 10^−6^ cm/s (34). In addition, the increased molecular weight of P4 compared to P0 may have contributed to its significantly reduced absorption rates. However, P8 exhibited the lowest absorption, despite having a lower molecular weight than P4. Unlike P8, which contained only large molecular weight fractions, P0 and P4 included smaller molecular weight fractions, resulting in their higher absorption rates. The increased absorption observed in P12 compared to P8 could be attributed to its decreased molecular weight due to the extended steaming.

In conclusion, while changes in molecular structure influenced by steaming duration do affect the absorption rates of PCPs, all PCPs demonstrated poor overall absorption. Unlike some polysaccharides that are well absorbed into systemic circulation, this suggests that the primary biological effects of PCPs may be mediated through modulation of the gut microbiota rather than direct systemic absorption.

### 3.4 Dynamic changes during *in vitro* fecal fermentation of PCPs

#### 3.4.1 Changes in pH value

**Figure 4:**
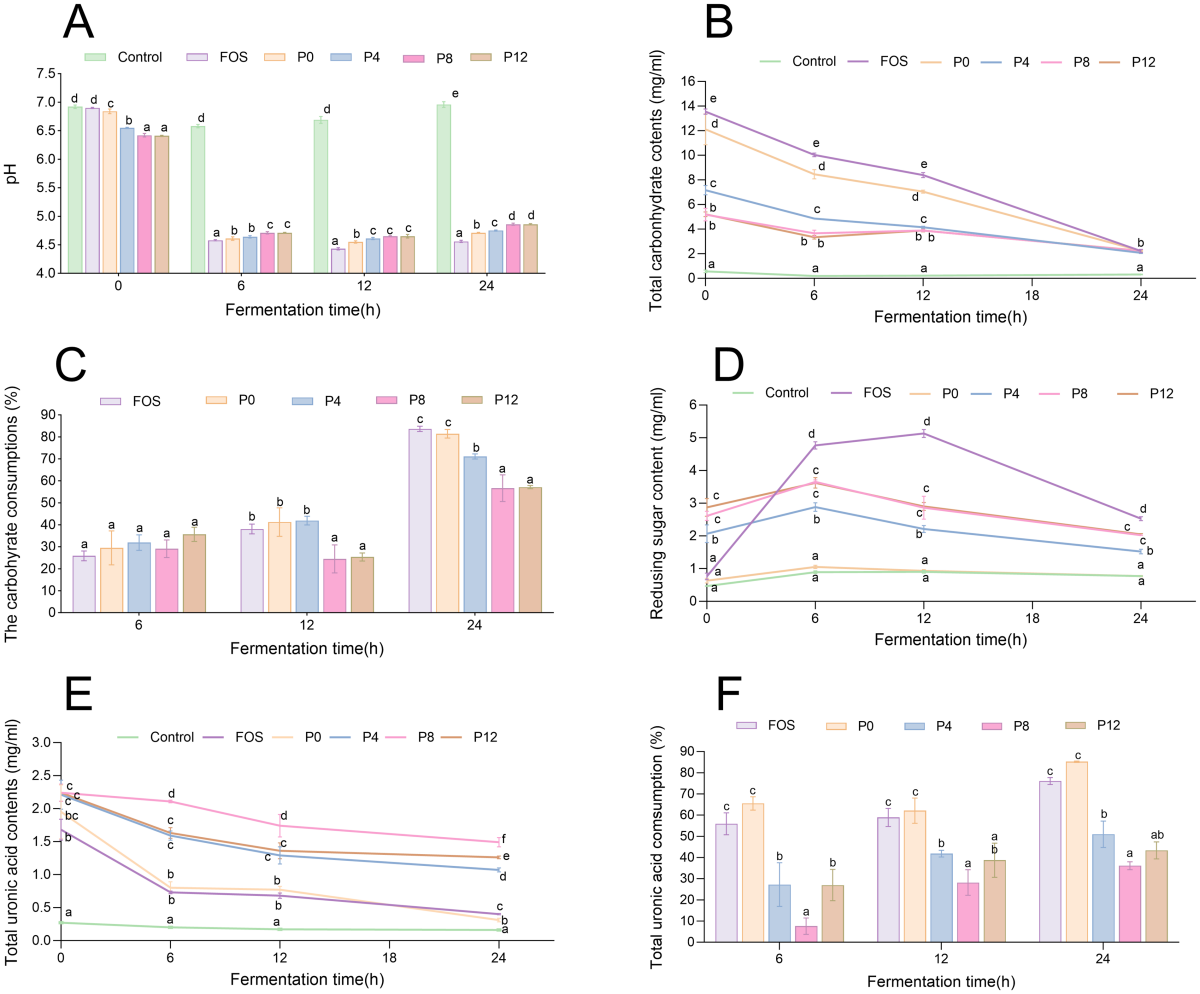
Dynamic changes in pH, carbohydrate, and uronic acid levels during *in vitro* fermentation of PCPs. **(A)** pH value changes over time. **(B)** Total carbohydrate content dynamics. **(C)** Carbohydrate consumption during fermentation. **(D)** Changes in reducing sugar contents. **(E)** Total uronic acid content dynamics. **(F)** Uronic acid consumption over time the different letters represent the statistical differences at *p* < 0.05 among different PCPs groups at same fermentation point.

Polysaccharides can be metabolized by gut microbiota, leading to the production of SCFAs and a decrease in pH. Figure 4A shows that the initial pH of PCP groups was lower than that of the control groups, likely due to the presence of uronic acid in PCPs (Figure 4E). The control group, lacking available carbohydrates, showed no significant pH variation after 24 h of fermentation. All PCP and FOS groups exhibited a rapid decline in pH within the first 6 h of fermentation, with the FOS group showing the lowest pH values at various fermentation times. The final pH values of FOS, P0, P4, P8, and P12 fermentation cultures decreased from 6.90 to 4.56 (*Δ* pH = 2.34), 8.84 to 4.71 (Δ pH = 2.13), 6.55 to 4.75 (Δ pH = 1.80), 6.42 to 4.86 (Δ pH = 1.56), and 6.41 to 4.86 (Δ pH = 1.55), respectively. This indicates that increasing the steaming duration resulted in higher pH values after 24 h of fermentation of PCPs, particularly for P8 and P12 from longer-steamed rhizomes, indicating less SCFAs production. Consistent with our findings that P0, containing the lowest molecular weight, resulted in the lowest final pH, degraded polysaccharides from longan pulp, litchi pulp, and blackberry with lower molecular weights were reported to produce a lower pH compared to the original polysaccharides with higher molecular weights (24, 47, 54). In addition, research has shown that the levels of glucose and xylose are significantly linked to pH reduction during fermentation (55). Consistent with our findings, extended steaming duration resulted in lower glucose and xylose levels in PCPs (21), potentially contributing to the lowest pH observed in P0. Thus, lower intestinal pH can promote the growth of beneficial bacteria while inhibiting harmful bacteria (54), suggesting that steaming duration could further influence the gut microbiota composition.

#### 3.4.2 Changes in carbohydrates

Carbohydrate consumption during fermentation indicates how effectively polysaccharides are utilized by gut microbiota. As shown in Figure 4B, FOS and all PCPs can be utilized by human gut microbiota, with no significant difference in total carbohydrate consumption among different groups after 6 h of fermentation (Figure 4C). However, after 24 h, the positive control FOS group showed the highest carbohydrate consumption at 83.6%. The P0 group had a carbohydrate utilization rate of 81.4%, higher than that of P4 (71.1%) after 24 h. The P8 and P12 groups exhibited lower utilization rates at 56.7 and 57.1%, respectively. Additionally, the P8 and P12 groups had significantly lower carbohydrate consumption at different fermentation time points compared to other groups. These results indicate that while PCPs can be utilized by gut microbiota, longer steaming duration reduce their utilization.

As shown in Figure 4D, reducing sugar content increased during the first 6 h, indicating the breakdown of glycosidic bonds in polysaccharides by gut microbiota. After this period, reducing sugar content decreased, likely due to microbial utilization. Steaming also increased the uronic acid content in the original PCPs (Figure 4E), consistent with previous findings (21), contributing the initial pH differences observed in Figure 4A. During fermentation, uronic acids were utilized by gut microbiota (Figure 4F). After 24 h, the utilization rate of uronic acids was significantly higher in the P0 group compared to PCPs from steamed rhizomes. Previous studies reported that high galacturonic acid content was associated with rapid utilization rates (56). However, the low consumption of uronic acid in steamed PCPs may be related to their triple-helical structures (21), which could reduce accessibility for gut microbiota.

In summary, the fermentation characteristics of PCPs are influenced by steaming duration, affecting pH changes, carbohydrate utilization, and uronic acid consumption by gut microbiota. The P0, containing lower molecular weight compared to PCPs from steamed rhizomes, led to a faster fermentation rate, higher carbohydrate utilization, and greater SCFA production. It is wildly recognized that low molecular weight or de-polymerized polysaccharides can be used to improve their bioactivity. Processing methods, such as ultrasonic degradation, that decrease molecular weight of polysaccharides, can accelerate fecal fermentation (57–59) and increase SCFA productions (50). But the molecular weight of polysaccharides are not proportional to its high prolife properties (60). The increase of steaming time (≥8 h) led to reduction of molecular weight, but the fermentation rate of P8 and P12 did not increased. In addition, the total carbohydrate consumption of different degraded polysaccharides were same at fermentation for 24 h (58). The low carbohydrate consumption of P8 and P12 may be attributed to their significant different monosaccharide composition compared to P0 and P4. It was found that monosaccharide composition in polysaccharides significantly affects the their utilization rate by intestinal flora and probiotic activity (22). Some neutral sugars, such as galactose, glucose, and xylose, are more easily fermented by microbiota than acidic sugars. Arabinose is utilized first, followed by glucose, fucose, and galacturonic acid (44, 61, 62). Thus, the increased steaming duration led to a decrease in glucose proportion in PCPs, correlating with the decreased fermentation rate from P0 to P12. It was confirmed that acidic monosaccharides are utilized later in the fermentation process (61), explaining the slower and lower utilization of PCPs from steamed rhziomes.

#### 3.4.3 Productions of SCFAs

**Figure 5:**
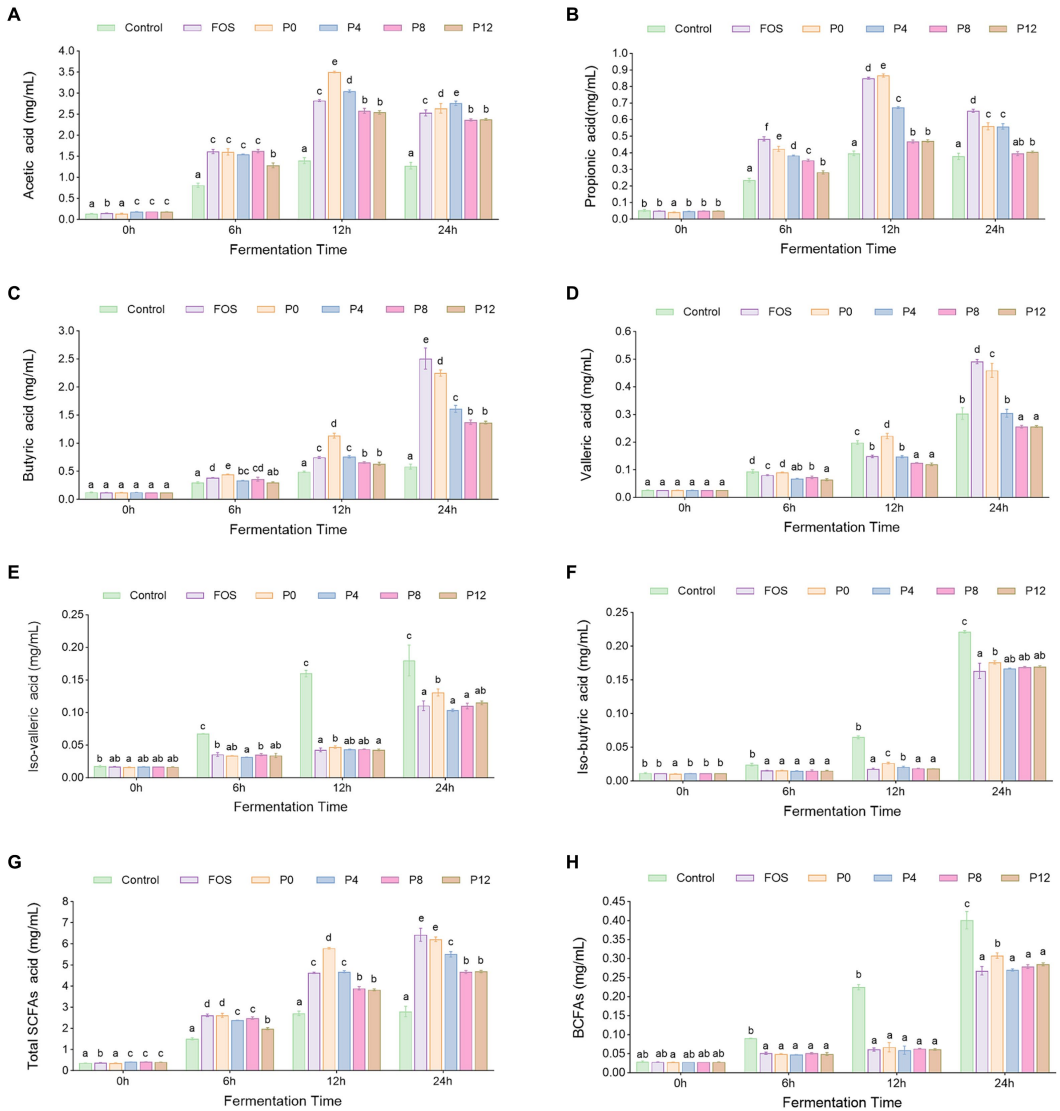
Dynamic changes of short chain fatty acids (SCFAs) contents s during *in vitro* fermentation of PCPs: **(A)** Acetic acids. **(B)** Propionic acids. **(C)** Butyric acids. **(D)** Valeric acids. **(E)** Isovaleric acids. **(F)** Isobutyric acid levels. **(G)** Total SCFAs concentration. **(H)** Branched short chain fatty acids (BCFAs) concentration. The different letters represent the statistical differences at *p* < 0.05 among different steaming groups at same fermentation point.

SCFAs are major metabolites produced by the gut microbiota during polysaccharide fermentation. The production levels of six types of SCFAs (acetic acid, propionic acid, butyric acid, valeric acid, iso-valeric acid, and iso-butyric acid) at different fermentation times are shown in Figures 5A–F. As shown in Figure 5G, the total SCFA levels gradually increased in all PCP groups, particularly within the first 12 h of fermentation. After 24 h, the P0 and FOS groups exhibited the highest total SCFAs production at 6.43 mg/mL and 6.22 mg/mL, respectively, compared with 2.80 mg/mL in control group, correlating with the pH decrease observed in these groups (Figure 4A). Conversely, the P8 and P12 groups showed the lowest total SCFAs production, both at 4.67 mg/mL (Figure 5G). In addition, P0 and P4 groups produced significantly higher amounts of acetic and propionic acid compared to P8 and P12 (Figures 5A,B). The lower molecular weight fractions in P0 and P4 (21) may have facilitated better degradation and utilization by gut microbiota, enhancing SCFAs production (24). Notably, P8 and P12 had same propionic acid production. P0 produced higher levels of butyric and valeric acids compared to the other groups (Figures 5C,D). The production of iso-butyric and iso-valeric acids, which are products of branched-chain amino acids, is shown in Figure 5H (63). All PCP and FOS groups had lower BCFA levels compared to the control group.

Consistent with our finding that P0 had highest SCFAs production, polysaccharides extracted by ultrasound- and enzyme-assisted extraction with low molecular weight have been shown to have a proliferative effect and stimulate higher SCFA output (44, 64). Monosaccharide composition affects both fermentation characteristics and SCFA production. Galacturonic acid was found to be negatively correlated with butyrate production, which aligns with our findings that P8 and P12, with higher galacturonic acid levels, exhibited lower butyrate production compared to P0 and P4 (61). However, it was found that galactose content in neutral polysaccharides has been associated with butyric acid production (61, 62), which does not align with our findings. In our study, PCPs from steamed rhizomes had higher galactose content but lower butyric acid production. This discrepancy may be due to the high proportion of acidic polysaccharides in PCPs from steamed rhizomes.

These results indicate that while PCPs can be metabolized by gut microbiota to produce SCFAs, prolonged steaming duration may diminish the fermentability of PCPs by gut microbiota.

#### 3.4.4 Impact of different PCPs on gut microbiota composition

**Figure 6:**
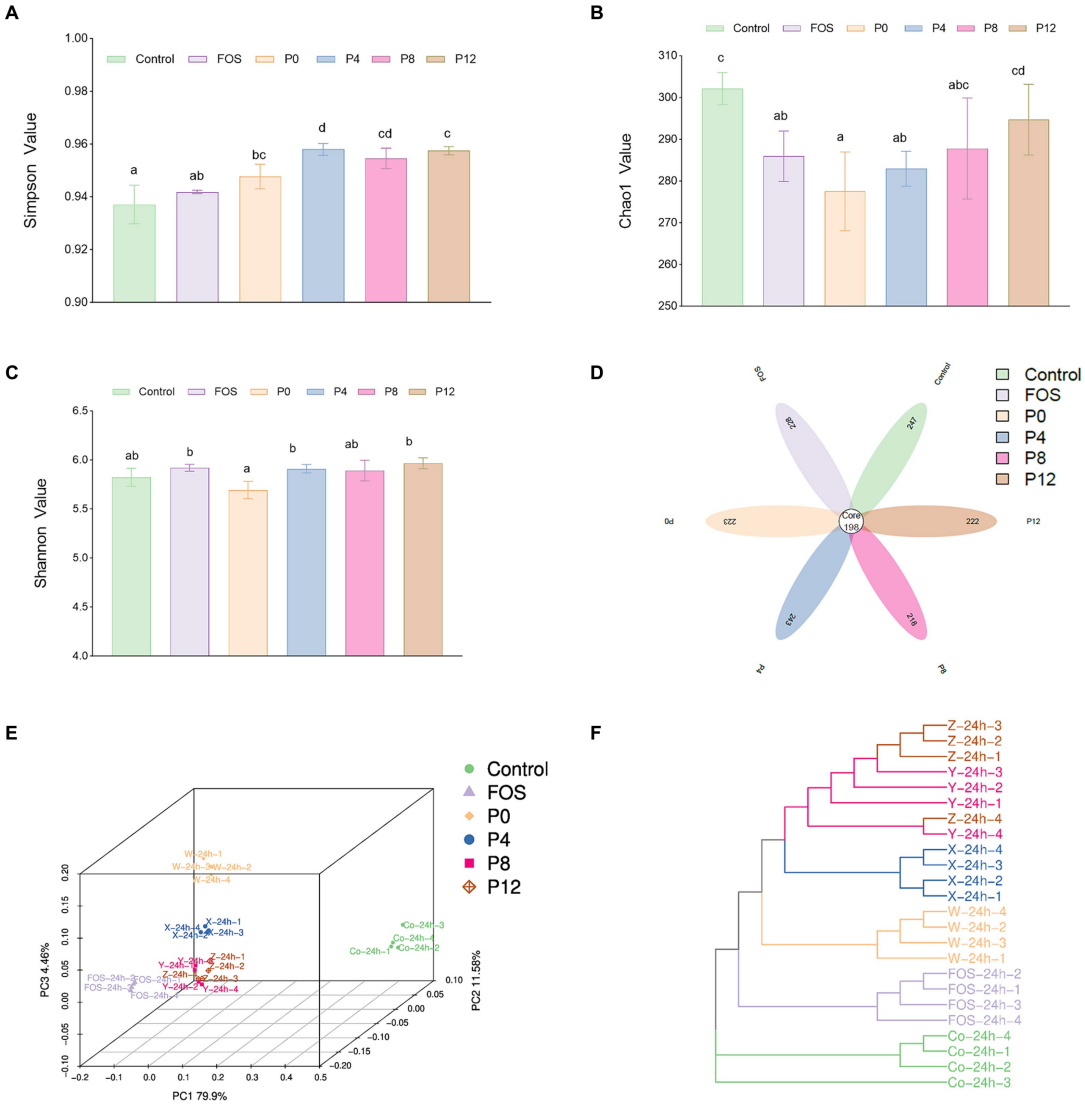
Impact of different PCPs on gut microbiota during *in vitro* fermentation: *α*-diversity: **(A)** Simpson index. **(B)** Chao1 index. **(C)** Shannon index; *β*-diversity. **(D)** Flower plot. **(E)** Principal coordinate analysis. **(F)** Bray Curtis phylogram tree. The different letters represent the statistical differences at *p* < 0.05 among different steaming groups.

The high-throughput sequencing technology of bacterial 16S rRNA was used to assess the impact of PCPs on gut microbiota composition. The *α*-diversity, which reflects the richness, diversity, and evenness of species within samples. Shannon and Simpson indexes represent the richness of community, while Chao1 indexes represent the richness of community (Figures 6A–D). All PCP supplements increased α-diversity compared with the control group, as indicated by Simpson indexes (Figure 6A). However, Shannon index indicated that PCPs and FOS did not significantly alter the diversity of the microbiota (Figure 6C). Furthermore, the Chao1 index showed a different trend compared to the Simpson index. The Chao1 values for P8 and P12 did not significantly differ from the control group, suggesting that longer steaming duration did not change the microbial diversity. In contrast, the P0, P4, and FOS groups had lower Chao1 values compared to the control (Figure 6B). Consistent with our findings, supplementation with polysaccharides from *P. cyrtonema* and five polysaccharides from sugar beet pulp did not alter α-diversity of gut microbiota (39, 61).

To further understand the impact of PCPs on gut microbiota composition, *β*-diversity was assessed. The β-diversity analysis indicates that the supplementation with PCPs and FOS significantly modifies the gut microbial community structure. The flower plot (Figure 6D) revealed 198 common amplicon sequence variants (ASVs) across all six groups. The number of unique ASVs was 247 in the control group, 228 in the FOS group, 223 in the P0 group, 243 in the P4 group, 218 in the P8 group, and 222 in the P12 group. Moreover, the principal coordinate analysis (PCoA) using Bray–Curtis dissimilarity (Figure 6E) showed distinct clustering of microbial communities between the control and treatment groups, suggesting that FOS and PCP supplementation significantly altered the gut microbiota. The PCoA plot demonstrated distinct aggregation patterns for the FOS, P0, and P4 groups, whereas the P8 and P12 groups exhibited similar microbial community structures. The Bray-Curtis phylogram tree (Figure 6F) confirmed these findings, with distinct branches for different groups, except for P8 and P12, which clustered closely together. These results indicate significant differences in microbial composition among the groups.

**Figure 7:**
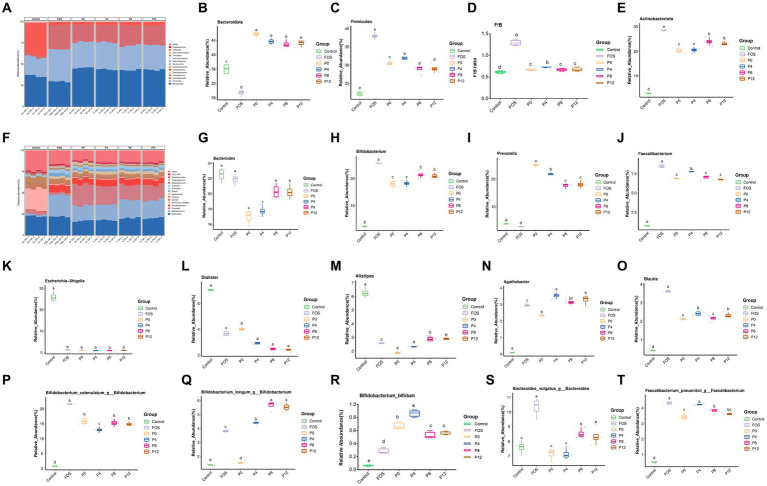
Effects of PCPs on gut microbiota community structure at phylum, genus, and species levels. Phylum level: **(A)** *Bacteroidota* **(B)**, *Firmicutes* **(C)**, ratio of *Firmicutes/Bacteroidota* **(D)**, *Actinobacteriota* **(E)**. Genus level: community structure at genus level **(F)**, *Bacteroides* **(G)**, *Bifidobacterium* **(H)**, *Prevotella* **(I)**, *Faecalibacterium* **(J)**, *Escherichia-Shigella* **(K)**, *Dialister* **(L)**, *Alistipes* **(M)**, *Agathobacter* **(P)**, *Blautia* **(O)**; Species level: *Bifidobacterium catenulatum* **(P)**, *Bifidobacterium longum* **(Q)**, *Bifidobacterium bifidum* **(R)**, *Bacteroides vulgatus* **(S)**, *Faecalibacterium prausnitzii* **(T)**. The different letters represent the statistical differences at *p* < 0.05 among different steaming groups.

In addition, the changes in gut microbiota composition due to PCP supplementation were analyzed at the phylum level, genus level, and species levels (Figure 7). The four PCPs significantly increased the abundance of Bacteroidota, Firmicutes, and Actinobacteriota, making them the dominant bacterial phyla (Figure 7A). As shown in Figure 7B, the relative abundance of Bacteroidota decreased in FOS group (29.41%) but increased in all PCP groups (42.94–45.60%) compared to the control (36.01%). The ratio of Firmicutes increased in FOS and all PCPs treated groups (28.85–37.94%) compared to the control (22.14%) (Figure 7C). Moreover, the FOS groups showed highest Firmicutes/Bacteroidota (F/B) (Figure 7D). The ratio of F/B in the P4 group was higher than in the other PCP groups. All PCP and FOS treatments significantly increased the proportion of Actinobacteriota (20.25–28.51%) compared to control (2.88%). However, P0 and P4 had lower abundance of Actinobacteriota compared to P8 and P12. All PCPs and FOS treatments significantly reduced the abundance of Proteobacteria, Desulfobacterota, and Fusobacteriota (Supplementary Table S1). Consistent with our findings, polysaccharides from *P. cyrtonema* increased the prevalence of Actinobacteria, which positively impact gut health (39), while certain pathogenic bacteria, such as Proteobacteria, were reduced by PCPs (39).

At genus level, the dominant microbiota included *Bacteroides, Bifidobacterium, Prevotella*, and *Faecalibacterium* (Figure 7F). As shown in Figures 7G,I, while the FOS treatment did not change the ratios of *Bacteroides* and *Prevotella,* all PCP treatments significantly reduced the ratio of *Bacteroide*s (16.97–20.30%) and increased the ratio of *Prevotella* of (17.64–24.99%) compared to control group (22.50 and 3.78%, respectively), respectively. After 24 h of fermentation, *Bifidobacterium*, which is a typical probiotic, became the dominant genus in FOS (25.72%) and all PCP groups (18.09–21.51%) compared to control groups of 2.29%. The relative abundance of *Faecalibacterium* also greatly increased in FOS (8.49%) and PCP groups (6.78–7.73%) compared with control (0.76%). Notably, the P0 group exhibited the highest relative abundance of *Prevotella* among all PCPs groups. Despite lower SCFAs production in the P8 and P12 groups (Figure 7G), these groups had a higher abundance of *Bifidobacterium* compared to P0 and P4. Moreover, Figures 6E,F show clustering analysis, indicating that P8 and P12 did not differ significantly in most bacterial genera, including *Bacteroides, Bifidobacterium, Prevotella, Faecalibacterium, Dialister*, and *Alistipes* (Supplementary Table S2). Similarly, P0 and P4 showed no significant differences in *Bacteroides, Bifidobacterium, Collinsella, Sutterella, Parabacteroides, UCG_002*, and *Lachnoclostridium* (Figures 7G–K; Supplementary Table S2). But P4 group showed advantageous abundances of *Faecalibacterium, Blautia, Agathobacter*, and *Lactobacillus* (Figures 7J,O; Supplementary Table S2). These genera are known for their beneficial effects on gut health. Previous studies have shown that high-fat diets increase the abundance of *Alistipes* and *Bacteroides*, while low-fat diets increase the abundance of *Faecalibacterium* and *Blautia* (65). All PCP treatments resulted in lower abundances of *Alistipes* and *Bacteroides*, and higher abundance of *Faecalibacterium and Blautia* indicating the potential of PCPs in treating gut microbiota disorders associated with high-fat diets. However, PCPs from longer steaming duration had higher abundances of these genera compared to P0 and P4. Notably, P0 had the lowest abundance of *Alistipes* among all PCP groups.

Additionally, the treatment with PCPs and FOS not only promoted beneficial bacteria but also inhibited the pathogenic bacteria. The pathogenic anaerobic bacterium, such as *Escherichia-Shigella* and *Dialister,* can disrupt the balance of intestinal flora (66, 67). All PCP and FOS treatments significantly reduced the abundance of *Escherichia-Shigella* and *Dialister* (Figures 7K,L). Especially, the abundance of *Dialister* was significantly lower in the PCP groups from steamed rhizomes compared to the P0 group. This indicates that steaming enhances the probiotic effects of PCPs by reducing pathogenic bacteria, thereby enhancing the protective effect against gut dysbiosis.

Furthermore, treatment with PCPs and FOS also had distinct effects at the species level. The top three *Bifidobacterium* at species level are shown in Figures 7P–R, FOS treatment resulted in the highest abundance of *Bifidobacterium catenulatum* (Figure 7P). The P4 group had a relatively lower abundance of *Bifidobacterium catenulatum* compared to the other PCP groups. PCPs from steamed rhizomes showed a higher abundance of *Bifidobacterium longum*, whereas the P0 group (raw rhizome) did not increase the abundance of this species (Figure 7Q), indicating that steaming enhances the ability of PCPs to promote *Bifidobacterium longum*. All PCP groups had a higher relative abundance of *Bifidobacterium bifidum* compared to the FOS group, with P4 group showing the highest relative abundance (Figure 7R), highlighting the effectiveness of PCPs in promoting this beneficial species. Moreover, the relative abundance of *Bacteroides vulgatus* increased in the P8 and P12 groups, while the P0 and P4 groups did not show a significant increase (Figure 7S). This suggests that longer steaming durations may facilitate the growth of *Bacteroides vulgatus.* The PCPs from steamed rhizomes showed a higher abundance of *Faecalibacterium prausnitzii* compared to the P0 group (Figure 7T), indicating that steaming enhances the ability of PCPs to support the growth of this beneficial bacterium.

Echoing our findings, polysaccharides from *Aspergillus cristatus* increased beneficial microbiota like *Prevotella* and *Faecalibacterium* while reducing harmful bacteria such as *Escherichia/Shigella* (68). Similarly, polysaccharides from sugar beet pulp promoted the growth of *Faecalibacterium* and *Bifidobacterium* (61) and *P. cyrtonema* polysaccharides increased *Prevotella* and decreased *Escherichia-Shigella* (39). Polysaccharides from litchi pulp, rich in arabinose, galacturonic acid, and galactose, favored the growth of *Bifidobacterium*, *Prevotella*, and *Bacteroides* (54). Blackberry polysaccharides significantly increased the abundance of *Bacteroides*, *Prevotella*, and *Blautia* (58).

Monosaccharides are the basic units of polysaccharides that target various microbiota (69). This research found that significant variations in PCPs caused by steaming influenced microbiota composition, attributed to the monosaccharide profiles altered by different steaming durations. Among all monosaccharides, galactose has the greatest impact on polysaccharide probiotic bioactivity (69). Galactose levels in PCPs gradually increased with longer steaming durations, with a notable rise in P8 and P12 (21). It has been reported that galactan can be utilized by gut *Bacteroides* (70), supporting our findings that increased steaming duration, which elevates galactose content in PCPs, also significantly increases the relative abundance of *Bacteroides*. Additionally, polysaccharides or oligosaccharides rich in galactose show selective stimulation toward *Bifidobacterium* (70, 71), corroborating our research that increased galactose in PCPs, due to longer steaming times, results in a higher abundance of *Bifidobacterium.* Moreover, galacturonic acid content is negatively correlated with *Dialister* (61), consistent with our study showing that increased galacturonic acid in PCPs, caused by steaming, led to significantly lower relative abundances of *Dialister* in P4, P8, and P12. Finally, xylose content in pectin has been found to correlate with *Blautia* abundance (72), consistent with our findings that P4 had both the highest xylose content in PCPs and the highest *Blautia* levels among all PCP groups. In addition, molecular weight plays a critical role in the selective modulation of gut microbiota (59). It was found that smaller molecular weight polysaccharides led to an increase in *Prevotella_9*, which aligns with our finding that P0, with the lowest molecular weight, had the highest proportion of *Prevotella* (24).

Furthermore, functional differences among the treatment groups were analyzed using PICRUSt2. Functional Composition Based on Clusters of Orthologous Groups of proteins (COG) revealed significant differences in the functional composition of gut microbiota between the PCP and FOS supplementation groups (Supplementary Figure S3). These differences indicate that each treatment uniquely influences the functional potential of gut microbiota. Analysis of functional Composition Based on Kyoto Encyclopedia of Genes and Genomes (KEGG) Pathways (Supplementary Figure S4) showed that all PCP and FOS supplements significantly altered the proportions of functional genes at levels 1 to 3. However, the specific changes in functional composition varied among the different treatments, indicating distinct functional impacts.

In conclusion, the present findings underscore the importance of processing techniques, which significantly alter the chemical composition in addition to structural changes of PCPs (21), leading to leading to distinct profiles in digestibility, absorption, fermentability, and selective modulation of gut microbiota with potential health benefits. Based on these results, it suggests that reducing steaming duration is crucial not only for preserving polysaccharide content but also for maximizing the absorption rate and probiotic activities of PCPs. Although the PCPs from raw rhizomes had the highest probiotic activities, consuming them directly can cause throat irritation. Therefore, utilizing PCPs as functional food ingredients is likely a more practical and effective approach (73). However, the structure–function relationship induced by steaming remains poorly understood, yet it is crucial for optimizing the health benefits of PCPs through precise steaming adjustments. Furthermore, this study found that PCPs are not fully digested or absorbed, making their interaction with gut microbiota pivotal to their physiological effects. Additional research is needed to clarify how different PCPs influence gut microbiota and its metabolites, and to elucidate the precise roles of various PCPs *in vivo* to validate their efficacy. For example, this study observed that P8 and P12 increased *Bifidobacterium* and *Bacteroides*, while P0 and P4 promoted *Prevotella*. Understanding how different combinations of gut microbiota altered by PCPs contribute to health promotion is essential.

In addition, while longer steaming durations are not beneficial for PCPs in terms of absorption and probiotic activities, commercial methods involving extended steaming of *P. cyrtonema* still dominate a significant market share. Future developments in functional foods from *P. cyrtonema* should consider the potential of flavonoids, polyphenols, and other components, as well as their interactions, in relation to the steaming process. Specifically, optimizing the steaming process to enhance or preserve the beneficial properties of these compounds could lead to more effective functional foods or supplements. Thus, thoroughly understanding the changes in different bioactive compounds in the rhizomes will provide a more comprehensive understanding of the regulatory effects of steaming duration on *P. cyrtonema*.

## 4 Conclusion

In summary, steaming *P. cyrtonema* rhizomes for varying durations produced different PCPs with distinct levels of total carbohydrates, uronic acid, protein, and polyphenols. These variations in chemical composition of the four PCPs suggest that steaming significantly alters the structural properties of PCPs, potentially influencing their digestibility, absorption, and fermentability. All PCPs were partially digestible under *in vitro* simulated saliva-gastrointestinal digestion conditions, with longer steaming durations increasing their digestibility. Despite poor absorption, indicating that their primary effects may be through modulating gut microbiota rather than direct absorption, all PCPs were degraded and utilized by human gut microbiota.

This steaming process significantly altered the microbial community composition, increasing the abundance of beneficial bacteria, and inhibiting pathogenic bacteria. Different PCPs had varying effects on gut microbiota composition and SCFAs production. Overall, the results demonstrate that the steaming duration of *P. cyrtonema* rhizomes plays a critical role in determining the digestive, absorptive, and fermentative properties of PCPs, ultimately affecting their probiotic potential.

## Data Availability

The original contributions presented in the study are included in the article/Supplementary material, further inquiries can be directed to the corresponding author.
