# Corrigendum: Various steaming durations alter digestion, absorption, and fermentation by human gut microbiota outcomes of *Polygonatum cyrtonema* Hua polysaccharides

**DOI:** 10.3389/fnut.2025.1561857

**Published:** 2025-02-17

**Authors:** Weijing Wu, Yanling Wang, Ping Yi, Xufeng Su, Yan Mi, Lanlan Wu, Qianglai Tan

**Affiliations:** ^1^Xiamen Medical College, Xiamen, China; ^2^Engineering Research Center of Natural Cosmeceuticals College of Fujian Province, Xiamen Medical College, Xiamen, China; ^3^Fujian Provincial Key Laboratory of Food Microbiology and Enzyme Engineering, Xiamen, China

**Keywords:** *Polygonatum cyrtonema* Hua polysaccharides, steaming, saliva-gastrointestinal digestion, absorption, *in vitro* fermentation, gut microbiota

In the published article, there was an error in Figure 4 as published. Due to an input error, the updated version of Figure 5 was incorrectly labelled as Figure 4, causing a duplication of Figure 4. The corrected Figure 4 and its caption appear below.

**Figure 4:**
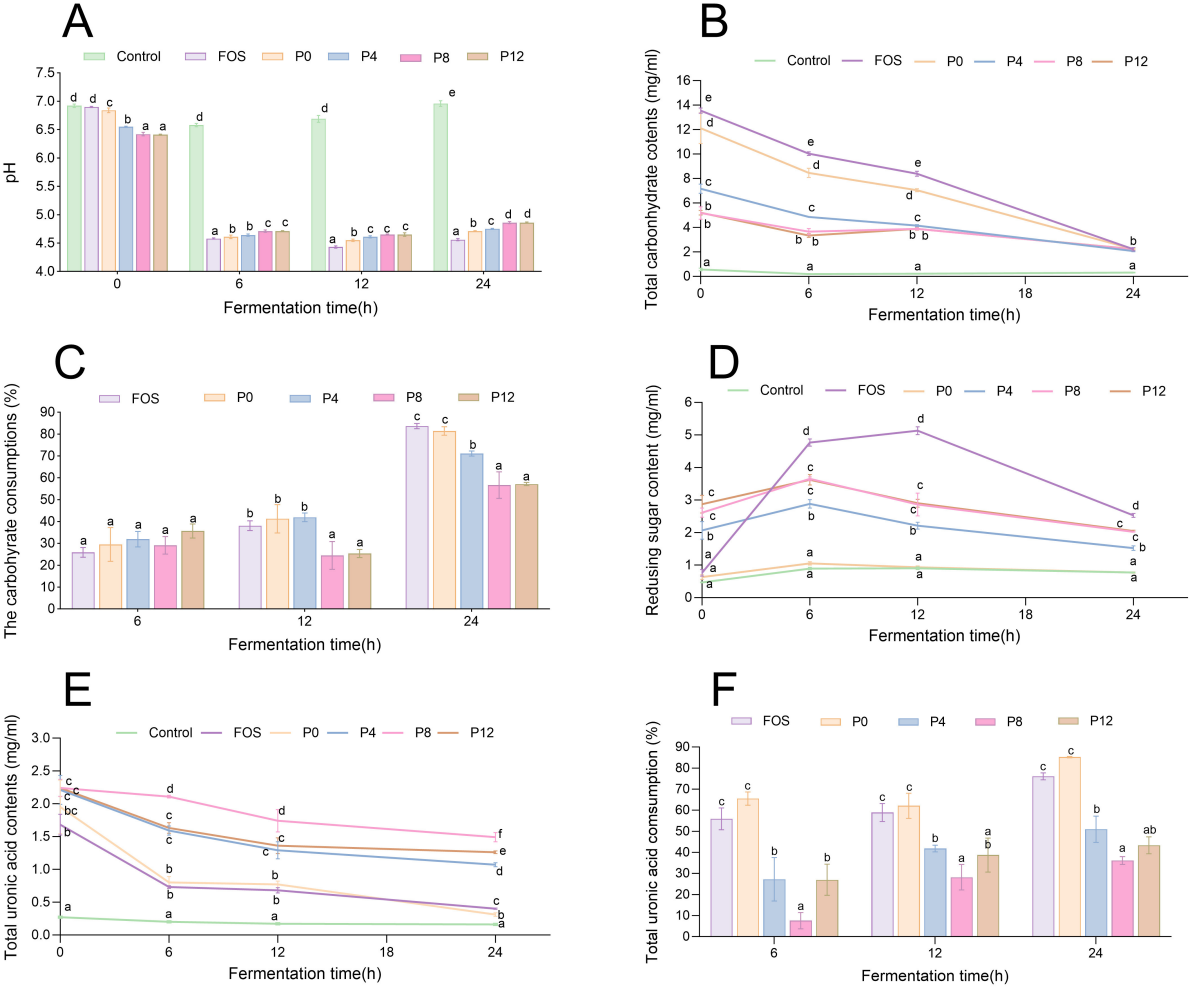
Dynamic changes in pH, carbohydrate, and uronic acid levels during *in vitro* fermentation of PCPs. **(A)** pH value changes over time. **(B)** Total carbohydrate content dynamics. **(C)** Carbohydrate consumption during fermentation. **(D)** Changes in reducing sugar contents. **(E)** Total uronic acid content dynamics. **(F)** Uronic acid consumption over time the different letters represent the statistical differences at *p* < 0.05 among different PCPs groups at same fermentation point.

The authors apologize for this error and state that this does not change the scientific conclusions of the article in any way. The original article has been updated.

